# Partial triceps tendon transfer for dynamic stabilization in posterolateral rotatory instability of the elbow: a biomechanical study

**DOI:** 10.1007/s00402-026-06520-z

**Published:** 2026-10-03

**Authors:** Christoph-Johannes Pucher, Fabian Lanzerath, Stephanie Lucina Kahmann, Michael Hackl, Lars Peter Müller, Tim Leschinger

**Affiliations:** 1 Centre of Orthopaedic and Trauma Surgery, University Medical Centre Mannheim, Mannheim, Germany; 2 Centre of Orthopaedic and Trauma Surgery, University Hospital Cologne, Cologne, Germany

**Keywords:** Posterolateral rotatory instability, Elbow instability, Triceps tendon transfer, Dynamic stabilization, Biomechanics

## Abstract

**Purpose:**

Posterolateral rotatory instability (PLRI) of the elbow results from lateral collateral ligament complex (LCLC) insufficiency. In the setting of failed conventional ligament reconstruction, no established salvage procedure is available. This study biomechanically evaluated partial triceps tendon transfer to the supinator crest as a dynamic stabilization concept for PLRI.

**Methods:**

Nine fresh-frozen human elbow specimens underwent cyclic external rotational loading of 0.5 Nm. Specimens were tested sequentially in the native state, an LCLC/common extensor origin-deficient unstable state, after transosseous LCLC repair, and after partial triceps tendon transfer to the supinator crest with the LCLC detached. Testing was performed under combined biceps/triceps loading conditions with external forearm rotation relative to the humerus as the primary outcome. A two-factor repeated-measures analysis of variance (RM-ANOVA) compared unstable, repaired, and tendon-transfer conditions across loading conditions. The native state was assessed unloaded and reported separately in a one-factor RM-ANOVA.

**Results:**

Intervention significantly affected external rotation (F(2,16) = 33.31, *p* < 0.001, η^2^_p_ = 0.81). Across loading conditions, partial triceps transfer significantly reduced external rotation compared to the unstable state (mean difference [MD]=-3.38°, *p* = 0.005), while LCLC repair showed lower external rotation than triceps tendon transfer (MD=-3.13°, *p* = 0.012). External rotation also differed significantly across the combined biceps/triceps loading conditions (Greenhouse–Geisser corrected F(1.26,10.11) = 77.00, *p* < 0.001, η^2^_p_ = 0.91), decreasing from 13.37° at 0 kg biceps/0 kg triceps to 2.42° at 0.5 kg biceps/4.0 kg triceps.

**Conclusion:**

Partial triceps tendon transfer improved dynamic elbow stabilization and significantly reduced PLRI in a LCLC‑deficient in vitro model. However, it did not fully restore joint stability, supporting its role as a potential adjunct or salvage option rather than a primary procedure. The findings highlight the importance of dynamic muscle tension in lateral elbow stability, while the time‑zero, in vitro design limits direct clinical translation and warrants further in vivo investigations.

**Supplementary Information:**

The online version contains supplementary material available at https://doi.org/10.1007/s00402-026-06520-z.

## Introduction

Posterolateral rotatory instability (PLRI) of the elbow results from insufficiency of the lateral collateral ligament complex (LCLC) [1, 2], with the lateral ulnar collateral ligament (LUCL) serving as the principal static restraint against posterolateral rotational moments [3–7]. Although anatomic ligament reconstruction remains the established gold standard for recurrent PLRI [8–12], conventional single- and double-strand techniques may be unfeasible in complex revision settings. Inadequate humeral bone stock – whether resulting from prior anchor placement, osseous defects, or failed reconstruction – may compromise secure graft fixation and can preclude standard techniques. (Fig. 7) Furthermore, recurrent instability remains the most common complication of LUCL reconstruction, occurring in approximately 8–15% of cases [13]. In these clinically demanding circumstances, alternative stabilization strategies are warranted.

Beyond static ligamentous reconstruction, biomechanical studies have increasingly emphasized the contribution of dynamic muscle tension to lateral elbow stability. The lateral triceps-anconeus complex has been identified as a relevant dynamic stabilizer capable of counteracting posterolateral rotational forces in LCLC-deficient elbows [14, 15]. These findings suggest that selective augmentation of dynamic muscular stabilizers may constitute a viable therapeutic concept, particularly when conventional static reconstruction is not feasible.

Building on this rationale, the present study biomechanically evaluated partial lateral triceps aponeurosis transfer to the supinator crest as a dynamic adjunct and potential salvage procedure for PLRI in the LCLC-deficient elbow. In the axial plane, the supinator crest lies at a greater radial distance from the center of external rotation than the lateral olecranon margin. Transfer of a lateral triceps strip to this insertion site is therefore expected to increase the triceps lever arm and redirect its force vector, thereby generating a larger internal moment opposing external rotation. We hypothesized that this configuration would enhance dynamic stabilization against PLRI and that its stabilizing effect would increase with triceps loading.

## Materials and methods

### Study design

This in vitro biomechanical study evaluated partial lateral triceps tendon transfer as a dynamic stabilization strategy in a fresh-frozen human elbow model of simulated posterolateral rotatory instability (PLRI). External forearm rotation relative to the humerus during cyclic external rotational loading constituted the primary outcome measure.

Each specimen underwent testing in the following fixed sequential order:

1. Native joint (baseline reference).
2. LCLC/common extensor origin (CEO)-deficient unstable state: PLRI model with LCLC/CEO detached from the lateral humeral epicondyle.
3. LCLC repair: transosseous locking suture repair of the detached LCLC/CEO.
4. Triceps tendon transfer: partial transfer of the lateral triceps aponeurosis to the supinator crest, performed after release of the repair, with the LCLC and CEO remaining detached from the lateral humeral epicondyle.

The fixed sequence was required by the progressive surgical preparation protocol: native baseline testing was followed by creation of the LCLC-deficient state, transosseous repair, release of the repair, and subsequent tendon transfer with potential impairment of the native triceps tendon and ulnar LUCL insertion. Consequently, randomization of intervention order was not feasible. Potential effects of repeated cyclic loading, tissue relaxation, and preceding surgical manipulation were minimized through standardized loading, a limited torque magnitude, and continuous saline irrigation.

Testing was performed under six predefined combined biceps/triceps loading conditions. The primary two-factor repeated-measures analysis included the unstable, repaired, and tendon-transfer conditions. The native state was assessed only under unloaded conditions and was used as a baseline reference.

**Fig. 1:**
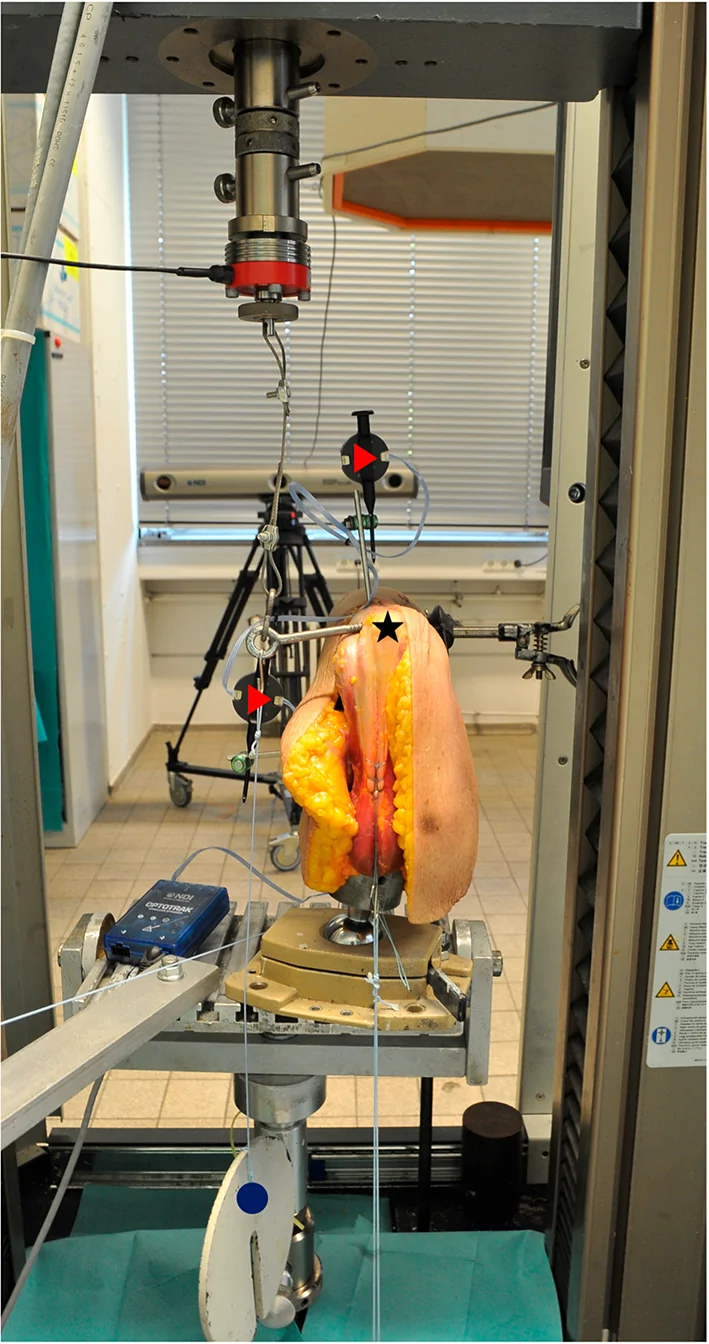
Test setup. Right donor elbow fixed to the custom-made testing rig in a servo hydraulic testing machine in 90° of flexion and full supination. A defined external torque of 0.5 Nm was applied cyclically to the proximal ulna via the testing machine pulling on an olecranon hook (black star). After the deflection the forearm was returned to its native starting position via a counterweight of 0.5 kg (blue circle). Biceps and triceps brachii muscles were slung and loaded with freely suspended weights to simulate dynamic stabilization of the joint through muscle tension (muscle preload). The movements of the ulna relative to the fixed humerus were tracked using optical markers (red triangles) and a 3D tracking system. [red triangles = optical markers; black star = olecranon with olecranon hook; blue circle = counterweight]

External forearm rotation was quantified using rigid intraosseous marker arrays and three-dimensional optical tracking (Fig. 1). The transfer condition was compared with the unstable and repaired states to quantify its dynamic stabilizing effect. We hypothesized that transfer of the lateral triceps aponeurosis to the supinator crest would reduce external rotation by increasing the triceps lever arm and redirecting the triceps force vector to oppose posterolateral rotational loading.

### Human elbow joint samples and preparation

#### Specimen data

Nine fresh-frozen human upper extremity specimens were included, reflecting limited specimen availability and sample sizes commonly used in comparable biomechanical studies of lateral elbow instability [9, 10, 16, 17]. 

All donors had provided written informed consent to the scientific use of their bodies during their lifetime. The study was approved by the Institutional Review Board of the Medical University of Cologne (approval number 22-1443). Mean donor age was 81.3 years (± 9 years; range 68–99 years). Two male and seven female specimens were examined (six left, three right extremities). Specimens were stored at − 20 °C and thawed at room temperature for 24 h before preparation. Radiographic and clinical screening excluded prior trauma, implants, and joint stiffness. Tissues were continuously irrigated with 0.9% saline.

#### Specimen preparation and test setup

The humerus was transsected to a total length of 15 cm. The proximal 2 cm were cleared of soft tissue and embedded in polymethylmethacrylate (Technovit 3040, Heraeus Kulzer, Germany). Following posterior exposure, Kocher’s interval was identified and a bicortical ulnar hole was created at the articular level for attachment of the olecranon hook for subsequent torque application (Fig. 1). Specimens were mounted in a custom testing rig within a servohydraulic materials testing machine (Z010, Zwick/Roell, Germany) at 90–100° of elbow flexion and full supination. Forearm motion was unconstrained and supported by a height-adjustable platform to avoid parasitic loading. Three-dimensional kinematics were recorded at 100 Hz using an optical tracking system (Optotrak Certus, Northern Digital Inc., Canada) with rigid intraosseous marker arrays on the humerus and ulna.

#### Experimental conditions

##### Unstable state (2)

Following baseline testing, the LCLC and CEO were detached from the lateral epicondyle through proximal extension of Kocher’s interval, reproducing the common humeral-sided LCLC rupture pattern [8, 18]. Measurements were repeated in this unstable configuration.

##### LCLC repair (3)

For LCLC repair, a 2.7 mm humeral tunnel was placed at the isometric center of the lateral humeral condyle. The detached ligament complex was reattached using a transosseous locking suture technique [19]. A No. 0 non-absorbable suture incorporated the LUCL, radial collateral ligament, and annular ligament, and fixation was secured with a sliding knot under 0.4 Nm internal rotation torque at 90° flexion and full pronation [20]. 

##### Partial triceps tendon transfer (4)

Before transfer, the transosseous LCLC repair suture was released and removed. The LCLC and CEO therefore remained detached from the lateral epicondyle, thereby recreating the intended LCLC-deficient configuration and allowing assessment of the isolated dynamic contribution of the tendon transfer.

**Fig. 2:**
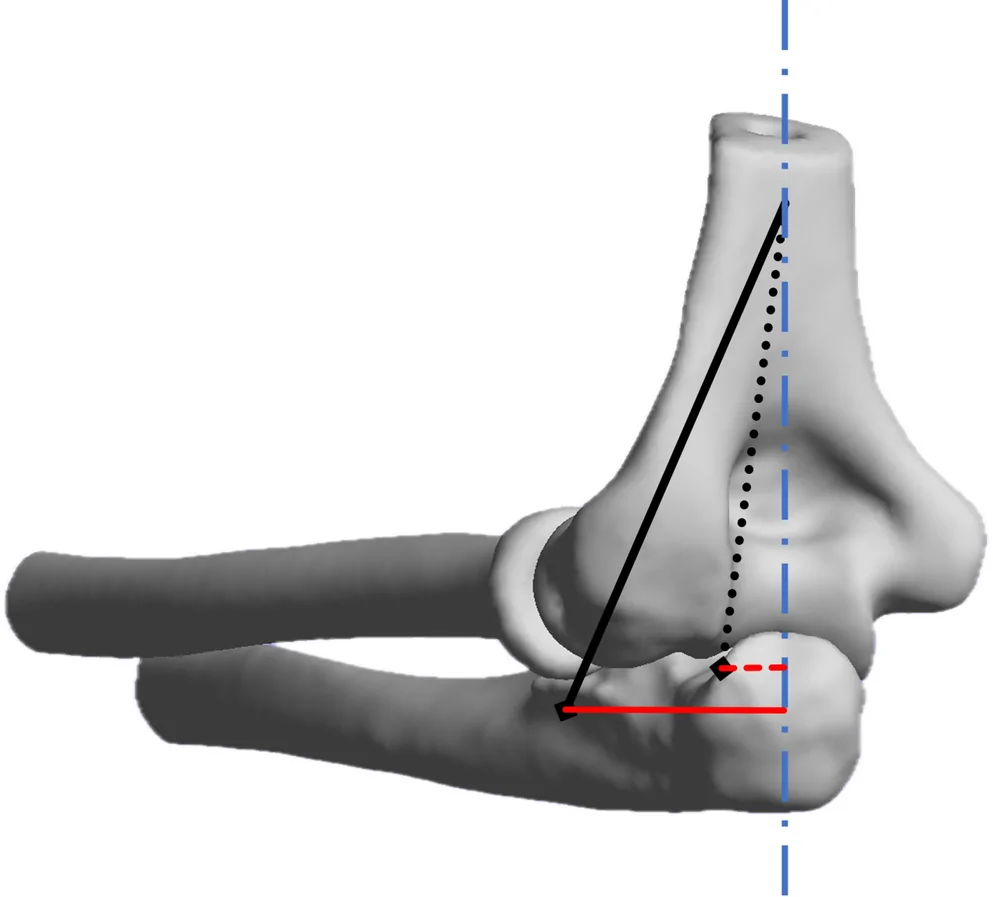
Biomechanics of the transfer. Smoothed 3D-Reconstruction of a left elbow joint in dorsolateral view created with 3D Slicer [29]. The geometry of the ulna in axial section indicates that the supinator crest has a greater radial distance to the center of the external rotation movement in PLRI than the lateral olecranon ridge, which is illustrated with the red lines. Consequently, the transfer (illustrated with the dotted and solid black lines) increases the lever arm (illustrated with the dotted and solid red lines) and the internal moment of the triceps muscle that counteracts the external rotation movement in PLRI. Depending on the joint position, the radial head acts as a hypomochlion and further extends the lever arm

The biomechanical rationale for the transfer was based on the anatomical relationship between the lateral olecranon margin and the supinator crest. In the axial plane, the supinator crest lies at a greater radial distance from the center of external rotation than the lateral olecranon margin (Fig. 2). Transfer of the lateral triceps aponeurosis to this location was therefore intended to increase the triceps moment arm and redirect its force vector to generate a larger internal moment opposing external rotation.

**Fig. 3:**
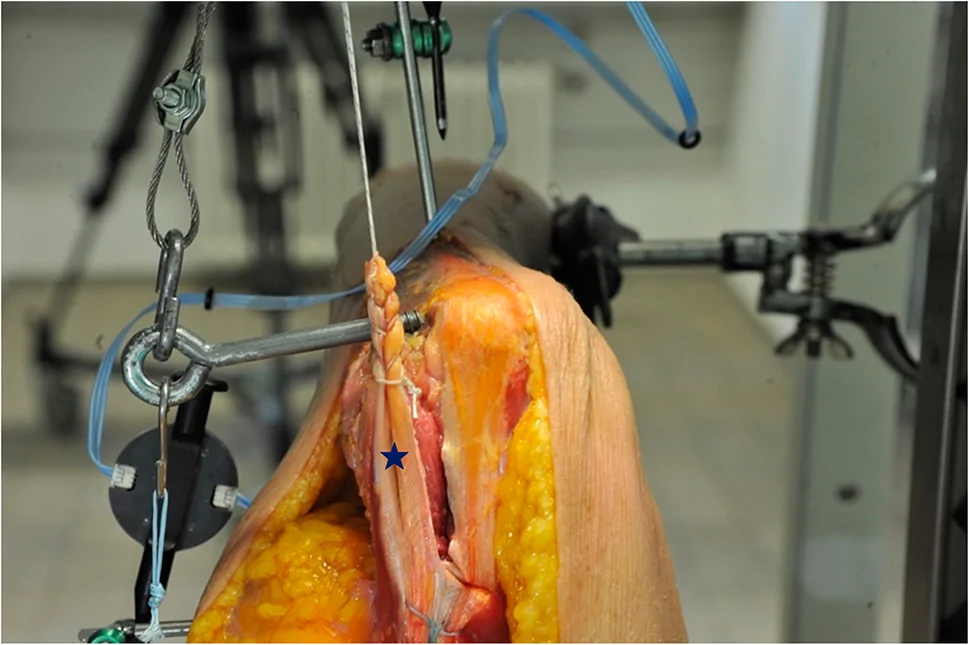
Preparation of the lateral part of the triceps aponeurosis. After releasing a portion of the muscle fibers attached to the distal humerus, the open end of the strip was whipstitched with No. 0 non-absorbable suture. [blue star = partially released triceps aponeurosis]

**Fig. 4:**
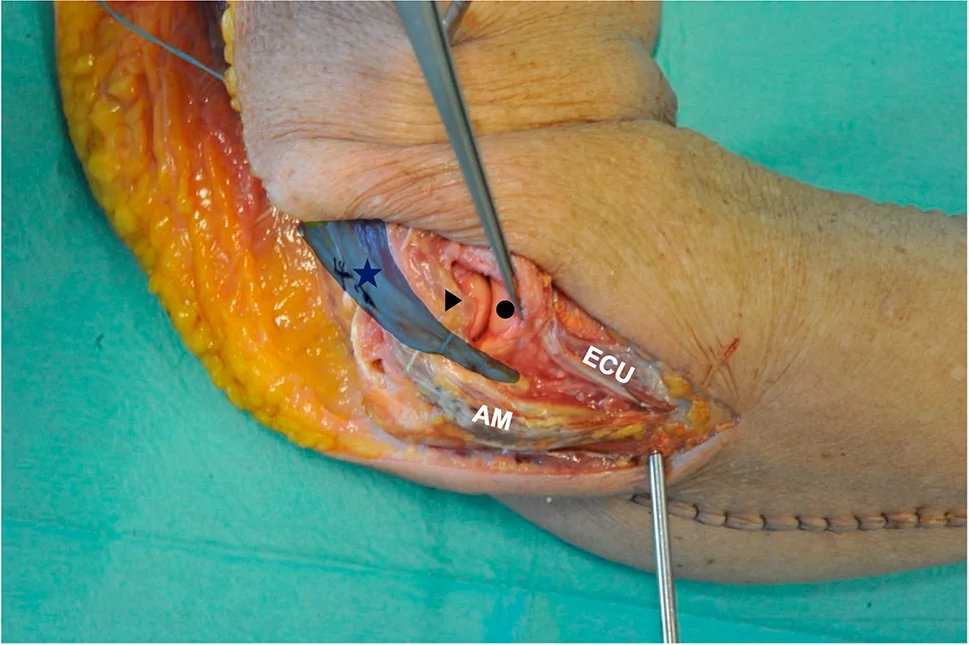
Right donor elbow with triceps transfer. The lateral part of the triceps aponeurosis (blue star) was shuttled between the fascia, the anconeus muscle, and the forearm extensor muscles towards the ulnar isometric center and fixed with a cortical button under slight internal rotational stress in manual tension. [blue star = transferred lateral triceps aponeurosis; blue circle = counterweight; black triangle = lateral Epicondyle; black circle = radial head; AM = anconeus muscle; ECU = extensor carpi ulnaris muscle]

For the transfer, a 1-cm-wide lateral strip of the triceps aponeurosis was mobilized, preserving proximal continuity. Additional length was obtained by partial release of distal muscle fibers. The free end was whipstitched with No. 0 non-absorbable suture. (Fig. 3). A 2.7 mm ulnar tunnel was created at the supinator crest isometric center, and the strip was routed to the new insertion site between the fascia, anconeus, and extensor musculature. Fixation was achieved with a cortical button under slight internal rotation and constant tension. (Fig. 4).

### Biomechanical testing

Cyclic external rotation torque of 0.5 Nm was applied to the proximal ulna by axial traction on the olecranon hook using the materials testing machine. The torque magnitude was selected to provide reproducible rotational laxity while avoiding soft-tissue failure during repeated testing, in contrast to protocols using higher torques [21]. Specimens were tested at 90° of elbow flexion and full supination. Forearm motion was unconstrained, and after each cycle the joint was returned toward neutral using a counterweight that generated 0.3 Nm of internal rotation torque (Fig. 1).

Dynamic muscle loading was simulated by applying freely suspended weights to the biceps and triceps brachii tendons along their physiological lines of action. Six predefined loading conditions were tested in the following order:

1. 0 kg biceps/0 kg triceps.
2. 0 kg biceps/0.5 kg triceps.
3. 0.5 kg biceps/0.5 kg triceps.
4. 0.5 kg biceps/1.0 kg triceps.
5. 0.5 kg biceps/1.5 kg triceps.
6. 0.5 kg biceps/4.0 kg triceps.

Each intervention-loading combination comprised 60 loading cycles at 7 N/s. External forearm rotation relative to the humerus was calculated as the mean value of the final 10 cycles of each run and served as the primary outcome measure.

### Statistical analysis

Statistical analyses were performed using IBM SPSS Statistics (version 29.0.1.1; IBM, Chicago, Illinois, USA).

The primary analysis was a two-factor repeated-measures analysis of variance (RM-ANOVA) with intervention (unstable joint, LCLC repair, and triceps tendon transfer) and combined biceps/triceps loading condition (six levels) as within-subject factors. External forearm rotation was the dependent variable. Because native-state measurements were available only at the unloaded baseline condition (0 kg biceps/0 kg triceps), the native state was excluded from the primary 3 × 6 repeated-measures model. A separate one-factor RM-ANOVA was performed at the unloaded baseline to compare the native, unstable, LCLC-repair, and triceps-transfer states.

Normality was assessed using the Shapiro-Wilk test. Sphericity was assessed using Mauchly’s test. Greenhouse-Geisser-corrected degrees of freedom and p-values were reported when sphericity was violated. Estimated marginal means were compared using Bonferroni-adjusted pairwise comparisons. Statistical significance was set at *p* < 0.05. Data are reported as mean ± standard deviation, unless otherwise stated.

## Results

External forearm rotation is presented as mean ± standard deviation (SD) for each intervention and predefined combined biceps/triceps loading condition in Table 1; Fig. 6. The primary two-factor RM-ANOVA included the unstable, LCLC-repair, and triceps-transfer states. Because native-state measurements were available only under unloaded conditions, the native state was evaluated in a separate one-factor RM-ANOVA at baseline.

**Table 1:** External forearm rotation for each intervention and predefined combined biceps/triceps loading condition.

Biceps / triceps loading [kg]	Native state, mean ± SD [°]	Unstable state, mean ± SD [°]	LCLC repair, mean ± SD [°]	Triceps transfer, mean ± SD [°]	Overall intervention effect	Unstable vs. LCLC repair, p	Unstable vs. triceps transfer, p	Triceps transfer vs. LCLC repair, p
0 / 0	4.81 ± 1.80	19.95 ± 5.02	6.00 ± 2.15	14.14 ± 6.23	F(3,24)=37.96,p<0.001ᵃ	<0.001ᶜ	0.058ᶜ	0.022ᶜ
0 / 0.5	-	18.18 ± 6.63	5.03 ± 1.91	10.78 ± 6.27	Roy’s largest root = 6.65; F(2,7)=23.28, p=0.001ᵇ	<0.001ᵈ	0.005ᵈ	0.037ᵈ
0.5 / 0.5	-	9.91 ± 4.35	4.21 ± 1.57	6.49 ± 2.75	Roy’s largest root = 3.03; F(2,7)=10.61, p=0.008ᵇ	0.004ᵈ	0.013ᵈ	0.014ᵈ
0.5 / 1.0	-	7.21 ± 3.10	3.72 ± 1.56	5.04 ± 2.09	Roy’s largest root = 3.19; F(2,7)=11.18, p=0.007ᵇ	0.004ᵈ	0.017ᵈ	0.007ᵈ
0.5 / 1.5	-	5.43 ± 2.30	3.26 ± 1.50	4.18 ± 1.87	Roy’s largest root = 2.94; F(2,7)=10.28, p=0.008ᵇ	0.005ᵈ	0.023ᵈ	0.011ᵈ
0.5 / 4.0	-	2.71 ± 1.42	2.09 ± 1.15	2.45 ± 1.15	Roy’s largest root = 2.73; F(2,7)=9.54, p=0.010ᵇ	0.016ᵈ	0.687ᵈ	0.065ᵈ

Values are reported as mean ± standard deviation (SD) in degrees. External forearm rotation was measured relative to the humerus. Loading conditions are given as biceps/triceps loads in kilograms

ᵃAt the unloaded baseline condition, a separate one-factor repeated-measures analysis of variance included all four intervention states: native, unstable, LCLC repair, and triceps tendon transfer. Mauchly’s test did not indicate a violation of sphericity (* p=0.153*). The overall intervention effect was significant: $\:F\left(3,24\right)=37.96$, * p<0.001*, $\:{\eta\:}_{p}^{2}=0.83$

ᵇAt the remaining five loading conditions, the reported multivariate simple effect of intervention was derived from the primary two-factor repeated-measures model including unstable state, LCLC repair, and triceps tendon transfer. The native state was not available beyond the unloaded baseline and was therefore excluded from this model

ᶜBonferroni-adjusted pairwise comparisons from the separate four-condition baseline analysis. The complete baseline pairwise comparisons were: native vs. unstable, * p<0.001*; native vs. LCLC repair, * p=0.985*; native vs. triceps transfer, * p=0.012*; unstable vs. LCLC repair, * p<0.001*; unstable vs. triceps transfer, * p=0.058*; and LCLC repair vs. triceps transfer, * p=0.022*

ᵈBonferroni-adjusted pairwise comparisons from the primary two-factor repeated-measures model

### Primary repeated-measures analysis

There was a significant main effect of intervention on external rotation, $\:F\left(2,16\right)=33.31$, * p<0.001*, $\:{\eta\:}_{p}^{2}=0.81$. Across loading conditions, estimated marginal mean external rotation was 10.56° (SE 1.20°) in the unstable state, 7.18° (SE 1.07°) after triceps tendon transfer, and 4.05° (SE 0.52°) after LCLC repair. Bonferroni-adjusted pairwise comparisons showed greater external rotation in the unstable state than after LCLC repair (MD 6.51°, * p<0.001*) and triceps tendon transfer (MD 3.38°, * p=0.005*). Triceps tendon transfer also showed greater external rotation than LCLC repair (MD 3.13°, * p=0.012*).

**Fig. 5:**
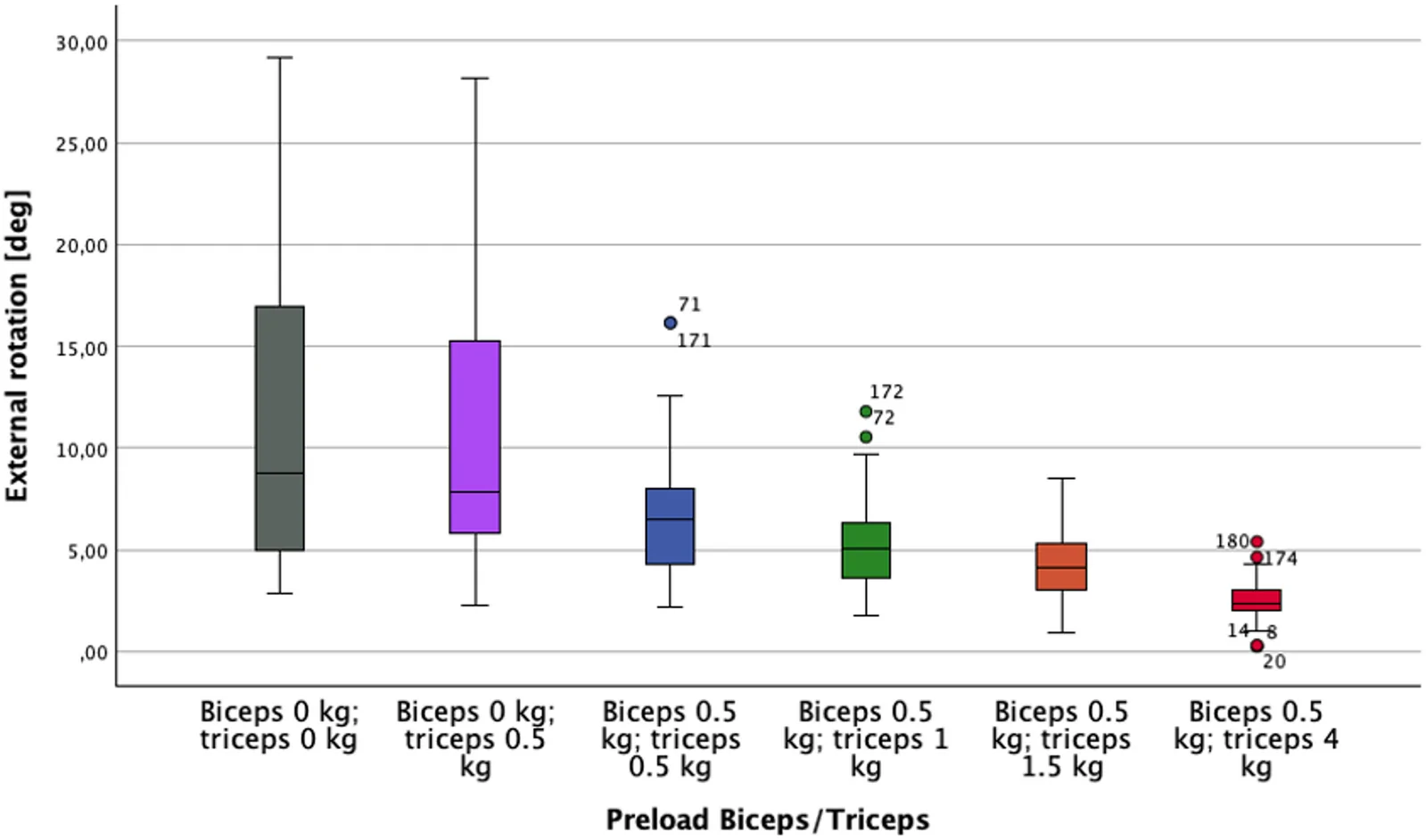
Boxplots of external rotation [deg] over all conditions across six predefined combined biceps/triceps loading conditions. [grey graph = 0 kg of triceps and 0 kg of biceps preload, purple graph = 0.5 kg of triceps and 0 kg of biceps preload, blue graph = 0.5 kg of triceps and 0.5 kg of biceps preload, green graph = 1 kg of triceps and 0.5 kg of biceps preload, orange graph = 1.5 kg of triceps and 0.5 kg of biceps preload, red graph = 4 kg of triceps and 0.5 kg of biceps preload]

**Fig. 6:**
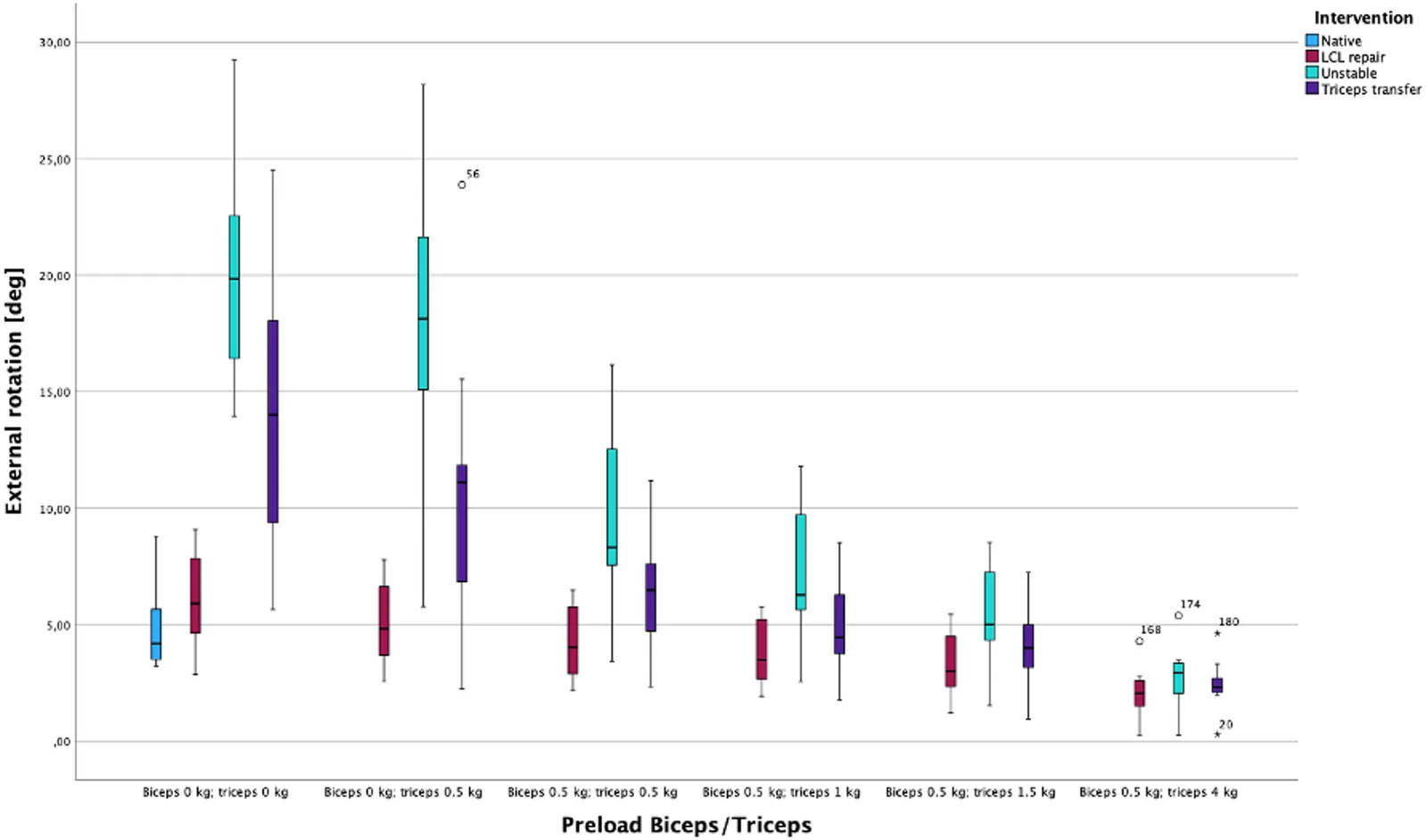
Boxplots of external rotation [deg] separated by the conditions native joint, unstable, LCLC repair, and triceps transfer across six predefined combined biceps/triceps loading conditions. [blue graph = native joint, red graph = LCLC repair, green graph = unstable joint, purple graph = triceps tendon transfer]

**Fig. 7:**
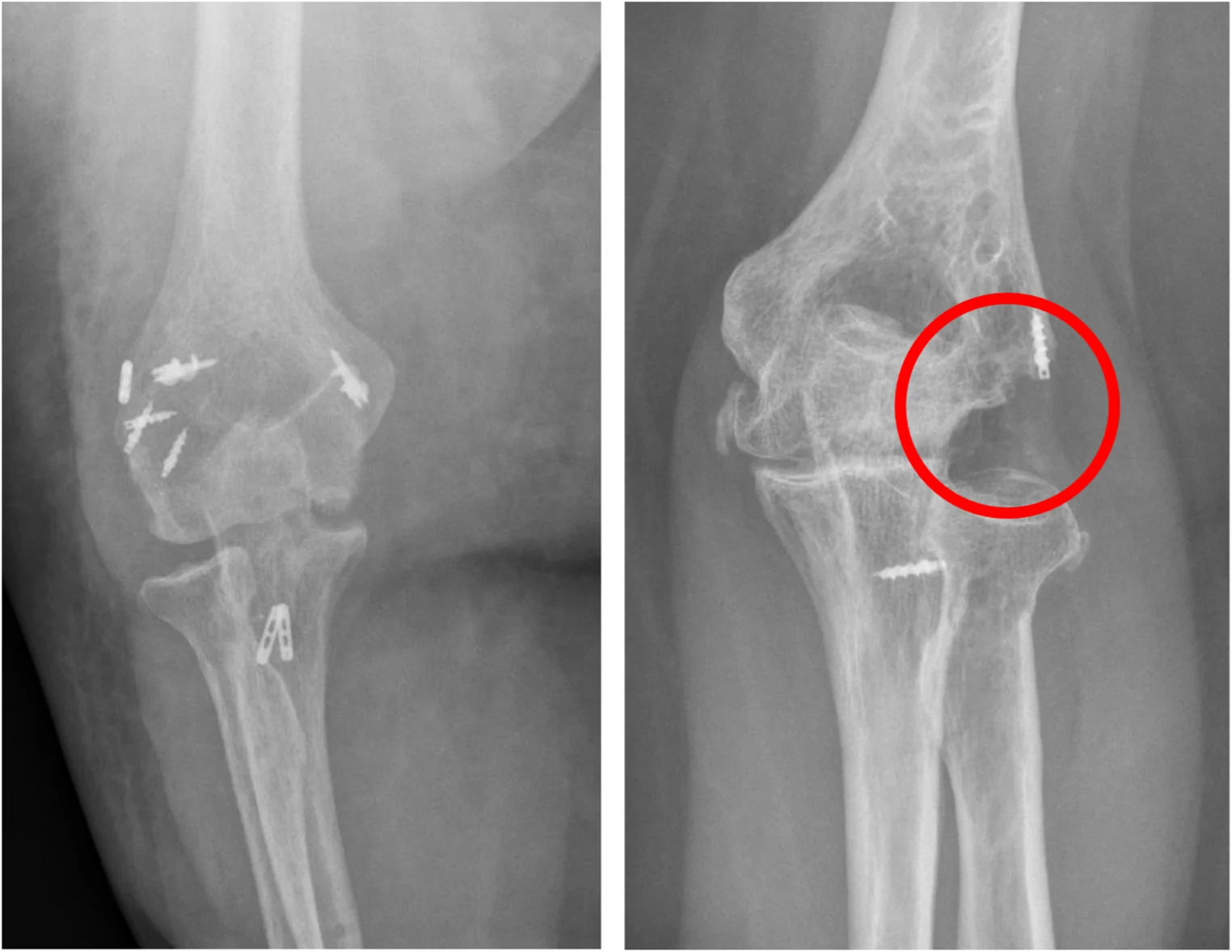
X-rays of cases requiring an alternative to conventional ligament reconstruction. Right side = Radiograph of a right elbow with multiple humeral suture anchors and two ulnar endobuttons; Left side = radiograph of a right elbow with a severe defect of the humerus

The predefined combined biceps/triceps loading condition significantly affected external rotation (Greenhouse-Geisser corrected $\:F\left(1.26,10.11\right)=77.00$, * p<0.001*, $\:{\eta\:}_{p}^{2}=0.91$). Across intervention states, estimated marginal mean external rotation decreased from 13.37° (SE 1.26°) at 0 kg biceps/0 kg triceps to 2.42° (SE 0.40°) at the highest loading condition 0.5 kg biceps/4.0 kg triceps (Figs. 5, 6, 7). All Bonferroni-adjusted pairwise comparisons between loading conditions were significant (all $\:p\le\:0.020$).

Furthermore, a significant intervention x loading-condition interaction was observed (Greenhouse-Geisser corrected $\:F\left(2.31,18.50\right)=24.45$, * p<0.001*, $\:{\eta\:}_{p}^{2}=0.75$).

### Unloaded baseline condition

At the unloaded baseline condition (0 kg biceps/0 kg triceps), external rotation was 4.81° ± 1.80° in the native state, 19.95° ± 5.02° in the unstable state, 6.00° ± 2.15° after LCLC repair, and 14.14° ± 6.23° after triceps tendon transfer.

The separate one-factor RM-ANOVA including all four baseline conditions showed a significant overall effect of intervention on external rotation ($\:F\left(3,24\right)=37.96$, * p<0.001*, $\:{\eta\:}_{p}^{2}=0.83$).

Bonferroni-adjusted pairwise comparisons demonstrated significantly greater external rotation in the unstable state than in the native state (MD, 15.14°; *p* < 0.001) and after LCLC repair (MD, 13.95°; *p* < 0.001). External rotation after triceps tendon transfer was significantly greater than in the native state (MD, 9.33°; *p* = 0.012) and after LCLC repair (MD, 8.14°; *p* = 0.022). In contrast, the native and LCLC-repair states did not differ significantly (MD, − 1.20°; *p* = 0.985). The difference between the unstable and triceps-transfer states did not reach statistical significance after Bonferroni adjustment (MD, 5.81°; *p* = 0.058).

### Highest loading condition

At the highest loading condition (0.5 kg biceps/4.0 kg triceps), the simple effect of intervention remained significant (Roy’s largest root = 2.73; $\:F\left(2,7\right)=9.54$; * p=0.010*). Mean external rotation was 2.71° ± 1.42° in the unstable state, 2.09° ± 1.15° after LCLC repair, and 2.45° ± 1.15° after triceps tendon transfer.

External rotation remained significantly greater in the unstable state than after LCLC repair (MD, 0.61°; 95% CI, 0.13° to 1.10°; *p* = 0.016). In contrast, comparisons between triceps tendon transfer and the unstable state (MD, − 0.26°; 95% CI, − 0.86° to 0.34°; *p* = 0.687) and between triceps tendon transfer and LCLC repair (MD, 0.35°; 95% CI, − 0.02° to 0.72°; *p* = 0.065) did not reach statistical significance.

## Discussion

This in vitro biomechanical study demonstrated a loading-dependent stabilizing effect of partial transfer of the lateral triceps aponeurosis to the supinator crest in a human donor elbow model of LCLC insufficiency. Across the predefined combined biceps/triceps loading conditions, partial triceps tendon transfer significantly reduced external forearm rotation relative to the unstable state, although it remained biomechanically inferior to transosseous LCLC repair. These findings support the central hypothesis that transfer of the lateral triceps aponeurosis to the supinator crest can augment dynamic resistance to external rotational loading by increasing the triceps moment arm and redirecting the resulting force vector against posterolateral rotational moments.

At 90° of elbow flexion, transection of the LCLC and common extensor origin produced a marked increase in external rotation. At the unloaded baseline, the separate one-factor repeated-measures analysis including the native state confirmed a significant effect of intervention. LCLC repair restored rotational behavior to a level not significantly different from the native reference state, whereas the unstable and tendon-transfer states demonstrated significantly greater external rotation than the native condition. In addition, external rotation after tendon transfer remained significantly greater than after LCLC repair. These results are consistent with the established role of the LCLC/LUCL as the principal static restraint against posterolateral rotational forces and with previous biomechanical investigations showing that lateral ligament repair or reconstruction can restore rotational stability [3, 7]. 

Across the primary three-condition repeated-measures model, the triceps tendon transfer consistently provided stability intermediate between LCLC repair and the unstable condition. The transfer reduced external rotation relative to the unstable state by a mean of 3.38° across the six predefined loading conditions, whereas LCLC repair showed a further mean reduction of 3.13° relative to tendon transfer. Thus, the transfer provided a measurable dynamic stabilizing contribution but did not reproduce the stabilizing capacity of an anatomic lateral ligament repair. This distinction is clinically relevant: the technique should be regarded as a dynamic supplement to, rather than a replacement for, restoration of the static lateral ligament complex.

At the highest combined loading condition (0.5 kg biceps/4.0 kg triceps), comparisons between tendon transfer and the unstable state, as well as between tendon transfer and LCLC repair, did not reach statistical significance after Bonferroni correction. However, the unstable and repaired states remained significantly different. Therefore, the numerical approximation of rotational values at the highest loading condition must not be interpreted as evidence of biomechanical equivalence or as proof that high muscle tension compensates for ligamentous insufficiency. Rather, the findings indicate that increasing combined biceps/triceps loading substantially reduced the observed differences in external rotation between the tendon-transfer condition and the comparator states.

The observed reduction in external rotation across loading conditions underscores the relevance of dynamic muscular forces for lateral elbow stability [14, 15, 21–23]. However, the predefined loading conditions did not constitute an isolated triceps dose-response protocol, because biceps loading was modified between selected test levels. The respective effects should consequently be interpreted as the response to combined biceps/triceps loading rather than attributed exclusively to increasing triceps tension. The maximum applied triceps load corresponded to approximately 39 N and was lower than estimates of triceps force during functional activities [24, 25]. Nevertheless, no direct inference regarding the magnitude of the transfer’s stabilizing effect under physiological loading is warranted. Physiological elbow stability depends on complex, position-dependent interactions among muscle co-contraction, joint compression, changing muscle-force vectors, passive capsuloligamentous restraints, and contributions from additional muscles, including the anconeus and forearm extensors [5, 6, 22, 26]. Accordingly, the present results demonstrate a dynamic stabilizing mechanism within a controlled time-zero cadaveric model, rather than providing a quantitative prediction of in vivo behavior.

From a clinical standpoint, the present data do not support partial triceps tendon transfer as a primary procedure for PLRI when anatomic LUCL reconstruction is feasible. The principal potential indication is a complex revision or salvage setting with recurrent PLRI in which conventional humeral-sided reconstruction is limited or precluded by deficient lateral humeral bone stock/bone loss, multiple prior anchors or bone tunnels, or failed previous reconstruction. In such situations, the procedure has potential advantages as it is independent of additional fixation at the lateral humeral epicondyle. By transferring an aponeurotic strip while preserving proximal continuity, the technique may provide a biological, dynamically acting stabilizing adjunct without requiring further humeral anchors or tunnels. Its potential clinical role is therefore as an adjunctive or salvage procedure in selected LCLC-deficient elbows, not as a standalone alternative to anatomic LUCL reconstruction.

Several limitations must be acknowledged when interpreting these results. The in vitro, time-zero design does not capture postoperative healing, graft integration, or neuromuscular adaptations, all of which are likely to influence clinical outcomes [9, 27]. Elbow stability was assessed only at 90° of flexion and full supination, a position of high susceptibility to PLRI [9, 17], so it remains uncertain whether the transfer provides sufficient stabilization throughout the full range of motion, particularly in functional extension and mid-flexion. The torque-controlled test setup employed a single, fixed loading axis, whereas in vivo joint loading is multiplanar and variable.

Furthermore, all specimens were evaluated in a fixed sequential order – native state, LCLC-deficient unstable state, LCLC repair, and tendon transfer. This order was required by the progressive surgical preparation protocol and could not be randomized. Although the applied torque, testing protocol, and specimen hydration were standardized to minimize potential bias, repeated cyclic loading, progressive tissue relaxation, and cumulative effects of preparation and repair may have influenced subsequent measurements. An order effect therefore cannot be excluded.

Before tendon transfer, the LCLC repair suture was released and removed to permit assessment of the transfer in an LCLC-deficient configuration. Although the LCLC and common extensor origin remained detached from the lateral epicondyle, the precise mechanical state present before the repair procedure may not have been fully restored. This potential confounding effect should be considered when interpreting the comparison between the initially measured unstable state and the transfer state.

Muscle simulation was limited to the biceps and triceps brachii. The anconeus and wrist/finger extensor muscles, which contribute to active lateral elbow stabilization, were not independently simulated [28]. In addition, the advanced donor age may have affected soft-tissue mechanical properties and limits extrapolation to younger and more active patient populations [16]. No failure testing was performed because each specimen was subjected to repeated loading under multiple experimental conditions. The applied torque was selected to generate reproducible instability while avoiding structural damage. Accordingly, absolute external-rotation values should be interpreted as relative biomechanical measures rather than clinical thresholds for instability.

Future studies should evaluate partial triceps tendon transfer across multiple elbow flexion angles and forearm-rotation positions under more comprehensive muscle loading conditions. Biomechanical assessment of transfer augmentation combined with standardized LUCL reconstruction may further clarify whether dynamic and static stabilization strategies provide complementary effects. Ultimately, prospective clinical studies are required to define tendon healing, durability, functional outcomes, complications, and the appropriate indication spectrum of this procedure in revision PLRI with compromised humeral-sided fixation.

## Conclusion

Partial triceps tendon transfer to the supinator crest improved dynamic stabilization and reduced external forearm rotation in this LCLC-deficient in vitro model. Nevertheless, in the setting of LCL insufficiency, it appears insufficient as a standalone procedure, as joint stability could not be fully restored. The translational relevance of these findings is inherently limited, as they reflect immediate, time-zero biomechanical effects without accounting for postoperative healing or long-term clinical outcomes. Partial triceps tendon transfer should therefore be considered a potential adjunct or salvage procedure for selected complex LCLC-deficient elbows in which conventional humeral-sided reconstruction is not feasible, rather than a primary treatment for PLRI. The time-zero in vitro design limits direct clinical extrapolation, and further biomechanical and clinical studies are required to define the procedure’s therapeutic role.

## Supplementary Information

Below is the link to the electronic supplementary material.

Supplementary Material 1

Supplementary Material 2

## Data Availability

The data that supports the findings of this study are available in the supplementary material of this article.

## Abbreviations

PLRI: Posterolateral rotatory instability
LCLC: Lateral collateral ligament complex
LUCL: Lateral ulnar collateral ligament
CEO: Common extensor origin
RCL: Radial collateral ligament
PMMA: Polymethyl methacrylate
ECU: Extensor carpi ulnaris
RM-ANOVA: Repeated-measures analysis of variance
Nm, kg, SD: Newton meter, kilograms, standard deviation
M: Mean values
SE: Standard error
